# Can computerized rumen mucosal colorimetry serve as an effective field test for managing subacute ruminal acidosis in feedlot cattle?

**DOI:** 10.1007/s11259-023-10231-w

**Published:** 2023-10-09

**Authors:** Nikolaos Voulgarakis, Dimitrios Gougoulis, Dimitra Psalla, Georgios Papakonstantinou, Panagiotis-Dimitrios Katsoulos, Konstantinos Katsoulis, Mariana Angelidou-Tsifida, Labrini Athanasiou, Vasilleios Papatsiros, Georgios Christodoulopoulos

**Affiliations:** 1 Clinical Veterinary Medicine Department, Faculty of Veterinary Science, University of Thessaly, Karditsa, Greece; 2 Laboratory of Pathology, School of Veterinary Medicine, Aristotle University of Thessaloniki, Thessaloniki, Macedonia, Greece; 3 Clinic of Farm Animals, Faculty of Veterinary Medicine, Aristotle University of Thessaloniki, Thessaloniki, Macedonia, Greece; 4 Department of Animal Husbandry and Nutrition, Faculty of Veterinary Science, School of Health Sciences, University of Thessaly, Karditsa, Greece; 5 Department of Animal Science, Agricultural University of Athens, Athens, Greece

**Keywords:** Subacute ruminal acidosis, Colorimetry, Rumen epithelium, Beef

## Abstract

Subacute ruminal acidosis (SARA) is one of the major nutritional disorders in the dairy and beef industries, leading to significant financial losses. Diagnosing SARA is challenging due to the need to evaluate multiple parameters, such as milk fat/protein ratio, ruminal lactate, and hemogram, instead of relying on a single definitive symptom or diagnostic method. This study aimed to evaluate the effectiveness of computerized rumen colorimetry in detecting SARA in beef cattle. Over one year, 75 cattle aged 8–10 months from five farms were periodically monitored for rumen pH prior to slaughter. Samples of rumen wall and rumen content were obtained at slaughter for analysis. The study found a positive correlation coefficient between rumen pH and color components, particularly for Red (0.853) and color lightness (L) (0.862). The darkening of the rumen epithelium’s color was attributed to the effect of rumen pH on the keratinized layer of the epithelium. Furthermore, an increase in the thickness of ruminal epithelium layers, particularly non-keratinized and total epithelium, was observed in animals with a history of SARA. It is possible that the lower rumen pH increases the rate of replacement of the keratinized epithelium, and the non-keratinized epithelium overgrows to compensate for the need to of produce keratinized layers. In conclusion, computerized rumen colorimetry shows promise as a reliable method for managing SARA in bovine farms by monitoring the condition in the slaughterhouse. Further research is needed to evaluate its effectiveness in detecting SARA in live animals.

## Introduction

High-concentrate diets find common usage in both dairy and beef industries to enhance production efficiency. Nonetheless, this practice can precipitate ruminal acidosis (RA) due to the rapid reduction of ruminal pH (Plaizier et al. 2012; Abaker et al. 2017). RA manifests as acute (ARA) or sub-acute (SARA) categories based on pH reduction and clinical manifestations (Aschenbach et al. 2011). Within intensive livestock farming, SARA is among the important nutritional disorders due to its potential for substantial welfare and economic repercussions. These encompass the deterioration of milk quality and quantity, diminished feed intake, weight loss, formation of liver abscesses, and elevated culling rates (Kleen et al. 2003; Krause and Oetzel 2006; Enemark 2008).

The task of diagnosing and confirming SARA proves to be intricate due to the absence of pathognomonic symptoms and precise diagnostic techniques (Nocek 1997; Tajik and Nazifi 2011; Snyder and Credille 2017). Field diagnosis of SARA primarily relies on measuring rumen fluid pH, whereby a rumen pH below 5.5–5.8 indicates the presence of SARA (Krause and Oetzel 2006; Plaizier et al. 2008; Humer et al. 2018). However, this pH measurement is subject to various influencing factors, including the method of measurement, sampling location, and timing of sampling (Neubauer et al. 2017; Villot et al. 2017).

Numerous researchers have noted a connection between the gray to dark discoloration of the rumen wall epithelium during necropsies of ruminants or culling and subacute and acute rumenitis (Enemark 2008). Nonetheless, an accurate interpretation of this coloration presents a challenge. Visual interpretation can be susceptible to bias, whereas instrumental colorimetric methods lack the capability for remote analysis and are particularly sensitive to the size of the colorimeter and spectrophotometer apertures (Yancey and Kropf 2008). Offering a practical and dependable alternative, computerized Red, Green, and Blue (RGB) analysis has found application in studies within the food industry (Chmiel et al. 2012; Silva et al. 2022).

This study seeks to establish a correlation between rumen epithelial color and SARA status in feedlot cattle, as determined through long-term periodic evaluations of rumen pH. The primary goal of this study was to assess the viability of computerized rumen colorimetry as a diagnostic tool for SARA within the context of herd management for feedlot cattle.

## Materials and methods

### Selection of farms

A total of 21 beef farms located in Thessaly, central Greece, were preselected for the study. These farms raise a breed of beef known as the “Greek Red Breed,“ an adapted variant of the Limousin breed. The final selection was based on their ration and three-year medical history, extracted from clinical case records at the Clinical Veterinary Medicine Department, Faculty of Veterinary Science, University of Thessaly. The 21 pre-selected beef farms were divided into 2 categories: farms with no previous history of ARA (2 farms) and farms with a history of ARA (19 farms). Among the latter, 3 subgroups were formed based on the percentage of digestive fiber in their ration: (i) 5 farms with less than 15% Neutral Detergent Fiber (NDF); (ii) 8 farms with 15–20% NDF; and (iii) 6 farms with 20–25% NDF. The study encompassed all farms with no prior history of ARA (Category I, 2 farms) and a random selection of 3 farms with a history of ARA from each of the 3 subgroups (Category II, 3 farms), using computer-generated random numbers. Table 1 presents a breakdown of the feed composition for the 5 farms included in the study. There were no modifications to the dietary composition over the course of the 12-month trial period.

**Table 1:** Ingredients, Chemical Composition, and Forage-to-Concentrate Ratio (F:C) in Total Mixed Rations for Farm 1 and Farm 2 (Category I), and Farms 3, 4, and 5 (Category II)

Item	Farm 1 Diet		Farm 2 Diet	Farm 3 Diet	Farm 4 Diet	Farm 5 Diet
Ingredients (% of DM)						
Alfalfa silage	20.0		15.0		20.0	19.0
Corn Silage	15.0		10.0	20.0	5.0	11.0
Wheat straw	5.0		15.0			
Ground Corn	25.0		20.0	60.0	40.0	40.0
Ground wheat				5.0		7.5
Ground barley	15.0		18.0		20.0	10.0
Soybean meal	12.0		14.5	12.0	12.5	10.0
Wheat Bran	5.0		5.0			
Vitamins and minerals	3.0		2.5	3.0	2.5	2.5
F:C	40:60		40:60	20:80	25:75	30:70
Chemical composition						
ME (Mcal/kg DM)	0.25		0.24	0.27	0.26	0.26
CP (% of DM)	16.1		15.2	14.0	16.3	15.2
NDF (% of DM)	31.9		34.4	14.8	22.2	23.2
ADF (% of DM)	18.3		20.7	9.1	12.2	12.9

ME = Metabolizable energy, CP = Crude Protein, NDF = Neutral Detergent Fiber, ADF = Acid Detergent Fiber

### Experimental design

In each of the 5 participating beef farms, 15 cattle aged 8–10 months were subject to periodic monitoring every 2 weeks for rumen pH over the course of 1 year before slaughter (from September 2021 to August 2022). Inclusion criteria encompassed a body weight range of 220–246 kg (with an initial body weight of 233 ± 13 kg, n = 75) and an absence of prior medical history. Measurement of rumen pH involved sampling the rumen content after esophageal tubing, specifically 3 h after the morning administration of concentrated feed. The average pH of all measurements taken throughout the year (24 measurements per animal) was designated as pH_farm_. Each animal was distinguished with distinct national registration numbers affixed to plastic ear-tags on both ears. All animals received uniform preventive medication, including vaccination and antiparasitic drugs.

The 15 monitored cattle on each farm were co-housed with an additional 4–12 cattle of the same age range (8–10 months at the onset of monitoring). All calves within these enclosures were slated for slaughter in September 2022, attaining an age of 20–22 months. During the slaughterhouse phase, samples of rumen wall and rumen fluid were procured from the monitored animals designated for regular production cycle slaughter. It is important to note that no animals were slaughtered exclusively for experimental purposes.

At the time of slaughter, all 75 monitored animals were sampled. Directly following evisceration, samples of rumen content and rumen wall were collected. The pH of rumen fluids was measured, the color of the rumen epithelium was visually assessed, and a digital gross image of the rumen wall epithelium was captured. The recorded pH measurement at this juncture was denoted as pH_abattoir_.

A total of 40 abdominal wall samples (20 from Category I and 20 from Category II) were submitted for histological examination at the Pathology Laboratory, Veterinary School, Aristotle University of Thessaloniki. The 40 rumen samples submitted were sourced from the initial 20 follow-up animals from each farm category sent to the slaughterhouse.

### Sample size justification

According to the literature, the range of SARA prevalence is relatively wide from 14 to 42% (Nagaraja and Lechtenberg 2007). In Greece, there is limited information about the prevalence of SARA (Kitkas et al. 2013). An average value of 22% prevalence was selected in the sample size calculation enhanced by the fact that animals were partly selected by farms with a previous history of ARA. Therefore, a sample size of 66 animals was calculated with precision 0.1 and confidence level of 95% to which 10% were added for any sample depreciation (Sergeant 2018). Based on this calculation, the 75 samples investigated in this study were considered an adequate sample size.

### Esophageal tubing (Rumen scoop)

Rumen fluid sampling was conducted using the Rumen Scoop (Flora, profs-products.com, Futtermittelüberwachung Bayern Regierung von Oberbayern, Sachgebiet 56, 80,534 München) (Geishauser et al. 2012). This all-metal device featured a 2.7-meter-long hose with a head and handle on each end. The head, functioning as a fluid scoop, was a cylindrical container measuring 3 cm in diameter and 21 cm in length, with a 40 mL capacity and side openings controlled by a screw mechanism.

To collect the rumen fluid, the head was inserted through the esophagus into the rumen for 10 to 20 s, allowing the fluid to be naturally drawn into the head without applying a vacuum. Subsequently, the handle activated the closing mechanism of the openings, and the device was carefully withdrawn. The head was then unscrewed and emptied. The gathered rumen fluid was immediately analyzed for pH using a digital portable pH meter (HI-2002 Edge pH Meter, Hanna instruments).

### Abattoir work protocol

Prior to slaughter, cattle were subjected to an approximate 3-hour fasting period, during which they underwent a comprehensive physical examination. They were marked with spray paint on the fleece for monitoring from slaughter to evisceration. After evisceration, each rumen was re-marked with a labeled cable before entering the gut room, where it was separated by slaughterhouse staff, following the procedure described by Jonsson et al. (2020).

The rumen fluid sample was obtained through aspiration, using a 16G 105-mm needle and a 50 mL syringe, from the eviscerated rumen. Subsequently, it was drained into a sterile glass container with a capacity of 100 mL. The pH of the rumen content was promptly measured using the same digital portable pH meter mentioned earlier (HI-2002 Edge pH Meter, Hanna instruments).

**Fig. 1:**
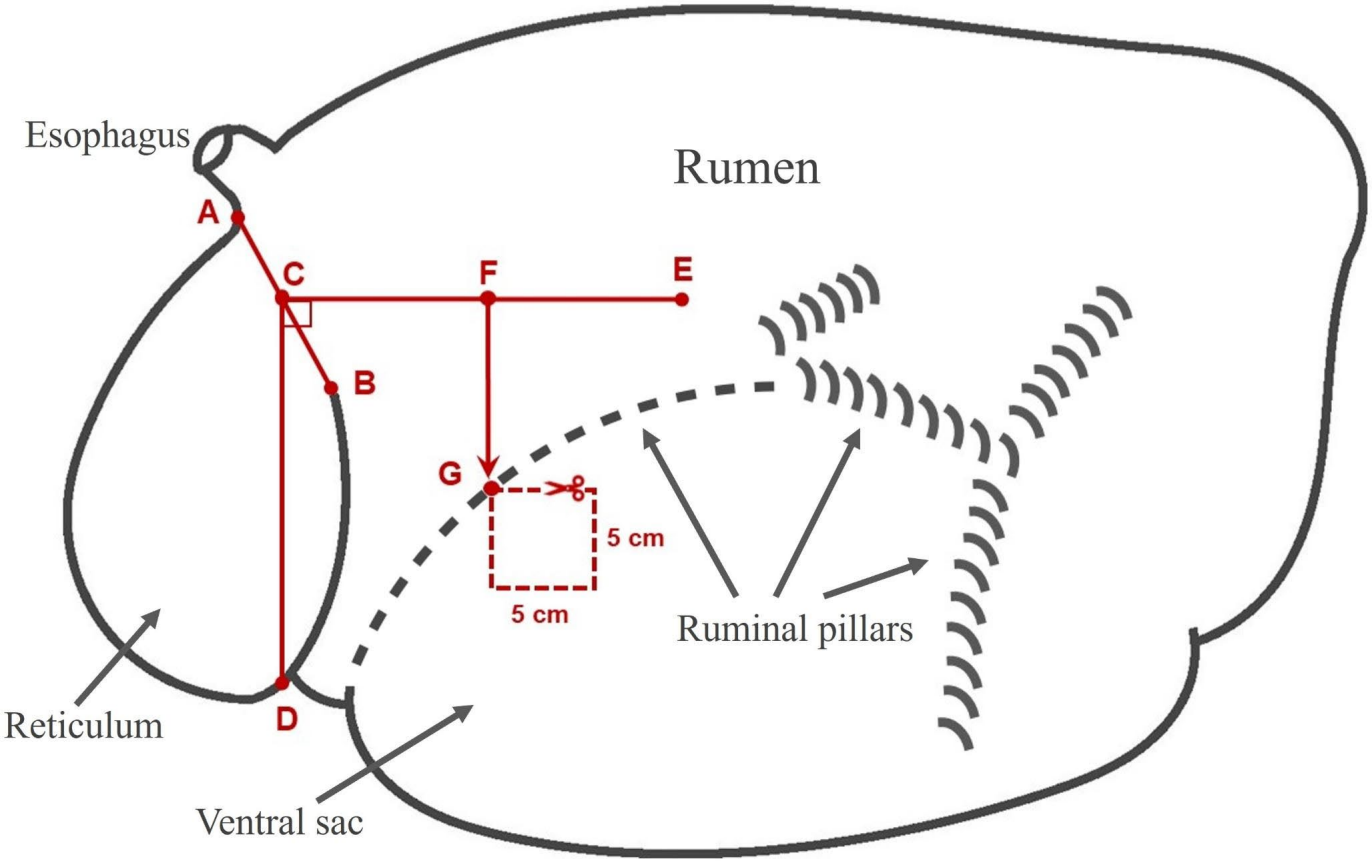
The procedure for sampling the rumen wall was performed following specific standard seven steps: (**i**) Commence by drawing a straight line between point A, the lower point of esophageal adhesion, and point B, the dorsal-caudal reticulorumen adhesion point. (**ii**) Locate the midpoint of line AB and designate it as point C. (**iii**) Draw a vertical line crossing point C and mark point D, where the vertical line intersects the edge of the reticulum. Measure the distance CD. (**iv**) Find point E in a manner that ensures the distance from C to E is equal to the distance from C to D and also ensures that angle DCE forms a right angle. (**v**) Identify the midpoint of line CE and label it as point F. (**vi**) Determine the point on the rumen pillar where a vertical line from point F intersects with the ventral pillar and mark it as point G. (**vii**) We now have the sampling region defined: it will be a square in the ventral ruminal sac, with the cranial side extending from the FG and point G as its upper-left corner (cranial-dorsal corner). The square should have dimensions of 5 cm x 5 cm

**Fig. 2:**
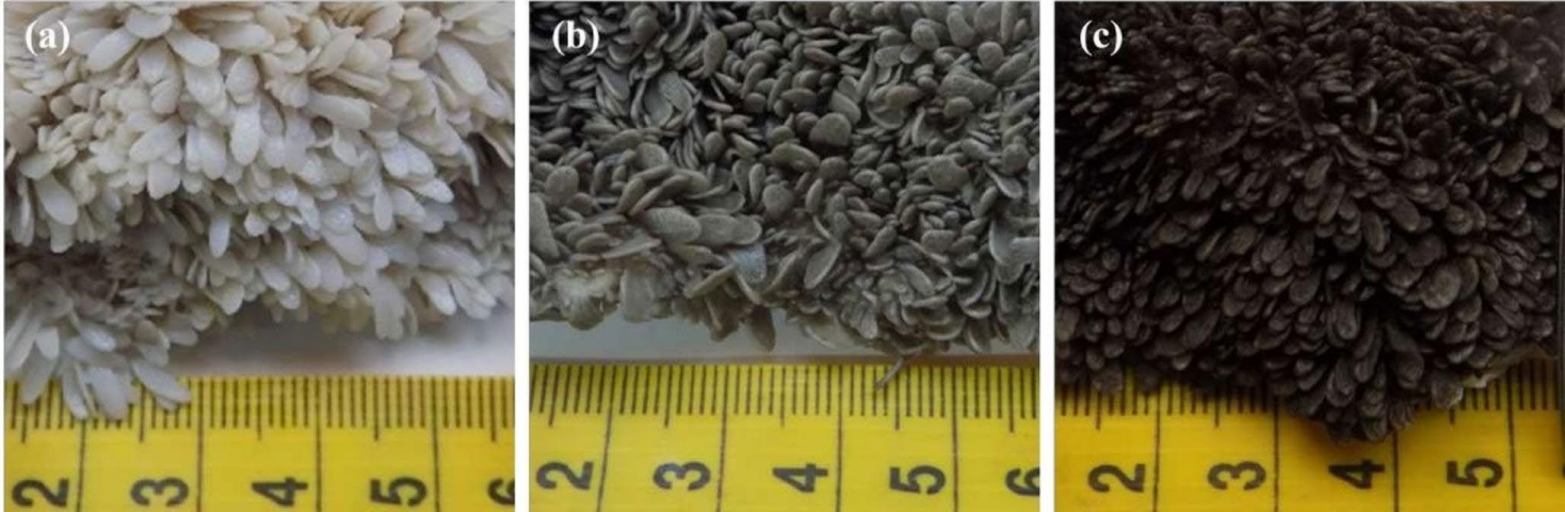
A three-tiered scale for visually assessing the rumen epithelium: (**a**) white/pale white (score 0); (**b**) grey (score 1); (**c**) dark grey or black (score 2)

The procedure for sampling the rumen wall involved specific steps. Firstly, the rumen was completely emptied of its contents. Next, the reticulorumen was oriented left-facing, with the reticulum in the front, the large rumen behind, the dorsal ruminal sac cranially, and the ventral ruminal sac caudally, with the esophagus positioned in front and dorsally. The location for the incision was determined based on standard measurements (Fig. 1), and a 5 × 5 cm square of the rumen wall was extracted for sampling. Subsequently, the sample was thoroughly washed with water to remove any visible residues of the rumen content. After cleaning, the sample epithelium was visually assessed, and was graded based on its color using a three-tiered score: (a) white or pale white (score 0), (b) grey (score 1), and (c) dark grey or black (score 2) (Fig. 2). Finally, a digital gross image was obtained following the standard procedure and the biopsy sample was placed in a container with a formalin solution for histological examination.

### Image acquisition and color measurement

For image acquisition, the collected samples were placed on a specific desk, and digital gross images were taken. A NIKON COOLPIX L330 digital camera with a 60-mm macro lens was used, with standard lighting conditions and settings. The images were saved in raw format.

The quantification of rumen discoloration was performed through software applications, namely RGB (RGB Color Detector 2022) and CIELab (CIE 1986). Within the RGB application, computations were made for color components encompassing Red, Green, and Blue (RGB), as well as Hue, Saturation, and Value (HSV), alongside Hue, Saturation, and Lightness (HSL). The determination of color components L (color lightness), a (redness-greenness of the color), and b (yellow-blueness of the color) was carried out utilizing CIELab (CIE 1986). For every rumen wall sample, the average values of these color components were computed from 9 distinct spot measurements, serving to facilitate subsequent statistical analysis. It is important to highlight that while the L, a, and b values are technically denoted as L*, a*, and b* respectively, for the sake of simplicity, this text omits the asterisks.

To distinguish between Category I and Category II, the color difference Delta E (ΔE) was calculated using the average values of L, a, and b color components. The following equation was used:

ΔE =$$\sqrt{{\left({L}_{I}^{}-{L}_{II}^{}\right)}^{2}+{\left({a}_{I}^{}-{a}_{II}^{}\right)}^{2}+{\left({b}_{I}^{}-{b}_{II}^{}\right)}^{2}}$$

Where ΔE = color difference; *L*_*I*_, *α*_*I*_, *b*_*I*_ = mean values of the color components determined for Category I, and *L*_*II*_, *a*_*II*_, *b*_*II*_ = mean values of the color components determined for Category II. ΔE is measured on a scale from 0 to 100, where 0 indicates less color difference, and 100 indicates complete distortion (Brainard 2003).

### Histological examination

**Fig. 3:**
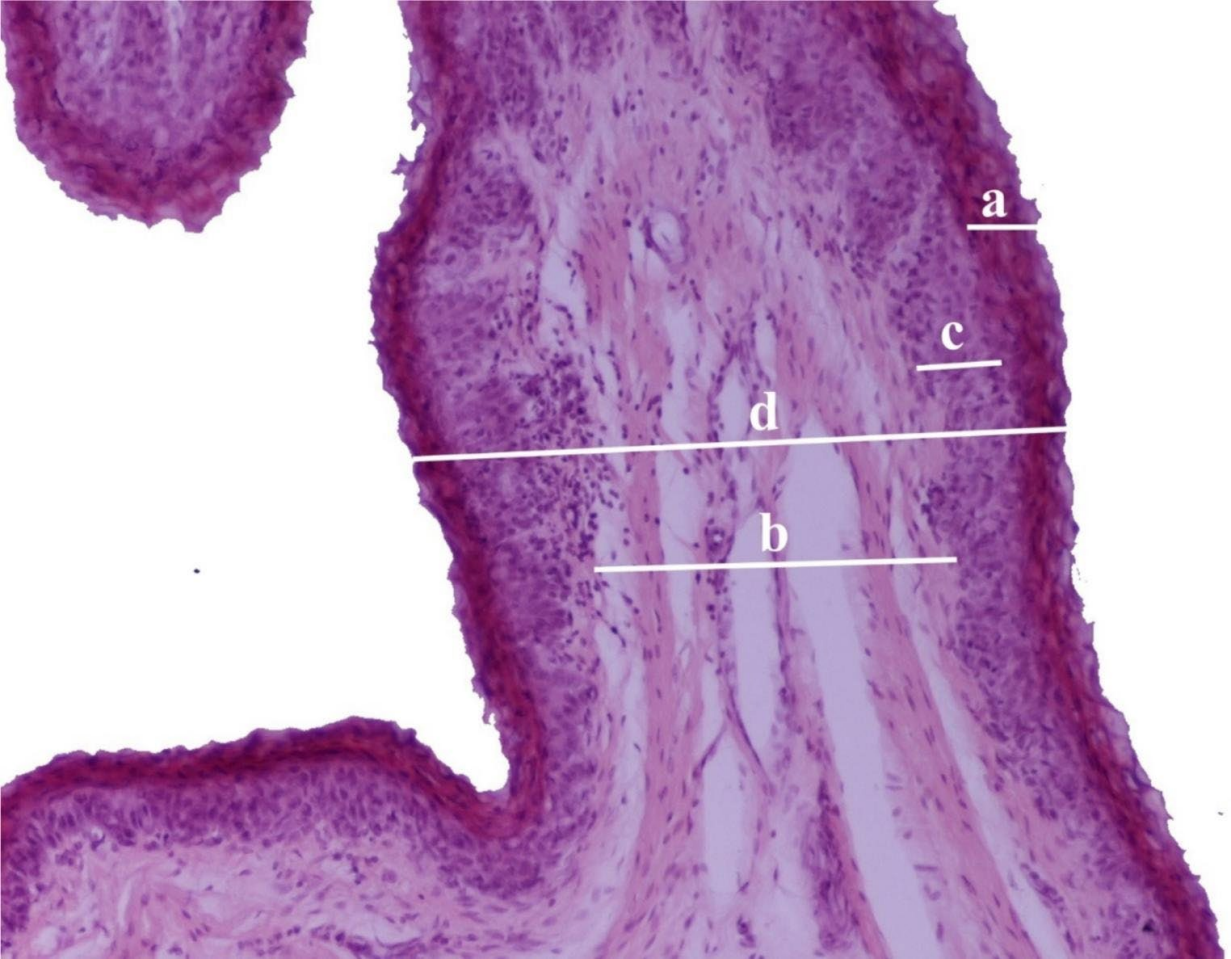
Measurements of the beef ruminal papillae: (**a**) Stratum corneum thickness. (**b**) Width of the connective tissue. (**c**) Thickness of the non-keratinized epithelium. (**d**) Total epithelium thickness. (Hematoxylin-eosin staining; magnification x10)

For histological examinations, the rumen wall samples were cut into 1 × 1 cm particles and fixed in 10% formalin buffer for 48 to 72 h. Afterward, they were dehydrated using alcohol solutions and routinely embedded in paraffin (Paraplast Plus®, Kendall, England). Dewaxed 3–5 μm-thick sections were stained with hematoxylin and eosin (H&E) for histopathological evaluation. From each sample, randomly selected fields with a x10 objective were captured with a microscope-coupled camera, and ImageJ software was used to measure stratum corneum thickness, connective tissue width, non-keratinized epithelium thickness, and total epithelium thickness in each photomicrograph (Fig. 3).

### Statistical analysis

Data on continuous variables were checked for normal distribution, and appropriate log transformations were applied to address skewness. A t-test was conducted to assess differences in pH values, color measurements, and rumen epithelium characteristics between the two animal categories. Correlations between pH values, color characteristics, and epithelium thickness were investigated through scatter plots and Pearson calculations (r). The mean pH status was calculated by averaging the measurements taken every 2 weeks for the continuously monitored animals. The chi-square test for independence, a non-parametric statistical procedure, was used to compare visual (categorical) data. Data analysis was performed using IBM SPSS Statistics version 29.0.0.0. (241), and statistical significance was set at either the 0.001 or 0.05 level.

## Results

All animals successfully completed the study, and prior to sampling at the slaughterhouse, all animals underwent clinical examination with no evidence of acute acidosis or other clinical disease recorded.

The monitored animals had a pH_farm_ range from 5.27 to 6.15, with a mean value of 5.80 (Standard Error [SE] = 0.020). Animals from farms with previous cases of ARA had a pH range of 5.27 to 5.90, with a mean value of 5.72 (SE = 0.023). In contrast, animals from farms without ARA cases had a pH range of 5.70 to 6.15, and their mean pH was 5.93 (SE = 0.021, p < 0.001).

Similarly, in the abattoir, the mean pH_abattoir_ value of Category I samples was 5.97 (SE = 0.023), which was statistically higher than the mean value of Category II samples (5.74, SE = 0.026, p < 0.001) (Table 2).

**Table 2:** Comparison of pH_farm_, pHabattoir, and color components (mean ± SD) between two categories of farms: Category I (without SARA) and Category II (with SARA) farms

	Category I (n = 30)	Category II (n = 45)	p value	95% Confidence Interval of the difference
pH_farm_	5.93 (0.12)	5.72 (0.15)	< 0.001***	0.14 to 0.28
pH_abattoir_	5.97 (0.13)	5.74 (0.18)	< 0.001***	0.16 to 0.30
L	48.1 (11.7)	29.62 (10.3)	< 0.001***	13.3 to 23.5
a	-2.9 (9.1)	0.55 (5.1)	0.420	0.13 to 6.7
b	14.5 (13.2)	0.77 (5.1)	< 0.001***	9.36 to 18.0
Red	118.1 (31.0)	71.4 (23.0)	< 0.001***	34.1 to 59.1
Green	115.3 (29.4)	70.1 (23.5)	< 0.001***	32.9 to 57.4
Blue	89.9 (33.8)	69.5 (23.8)	0.003*	7.2 to 33.7
RGB-SUM	323.3 (85.0)	211.1 (69.0)	< 0.001***	76.6 to 147.8
HSV	20.1 (26.6)	19.9 (16.4)	0.968	9.7 to 10.1
HSL	40.1 (13.5)	25.6 (11.5)	< 0.001***	8.7 to 20.3

*Significant difference at p ≤ 0.05

***Significant difference at p ≤ 0.001

L: color lightness

a: redness-greenness of the color

b: yellow-blueness of the color

HSV: Hue, Saturation and Value

HSL: Hue, Saturation, and Lightness

### Color measurements

The chi-square test for visual examination showed significant differences, with a higher percentage of grey and black discoloration in Category II compared to Category I (p = 0.044) (Table 3).

**Table 3:** Comparison of the visual examination results between Category I (Farms without SARA) and Category II (Farms with SARA)[number of samples (percentage)]

	Category I (n = 30)	Category II (n = 45)	p value	Pearson Chi-Square
White/Pale White	27 (90.0%)	29 (64.4%)	0.044*	6.225
Grey	2 (6.7%)	10 (22.2%)		
Dark Grey/Black	1 (3.3%)	6 (13.3%)		

*Significant difference at p ≤ 0.05

The average value of the L component in Category I samples was 48.1 units (SE = 2.14), which was significantly (p < 0.001) higher than the value for Category II (29.6 units, SE = 1.53); a similar trend was observed for the b component. However, there was no significant difference in the average value of the a component (Table 2).

The color difference (ΔE) between Category I and Category II rumen wall samples was calculated using the L, a, and b color components. A significant color difference (ΔE = ~ 13.2177) was found between the 2 categories (Table 2).

Concerning the color measurement using the RGB Analysis system, Category I samples exhibited significantly higher mean values for Red, Green, Blue, and RGB-SUM in comparison to Category II samples. Additionally, significant differences were observed for the L value, but not for the V value (Table 2).

### Ruminal epithelium histopathology and histomorphometry

Histological examinations showed no pathological findings in the rumen epithelium samples, and there were no signs of parakeratosis. In samples with the darkest coloration, the dark color was limited to the keratinized layer.

Based on the results, Category II exhibited significantly greater thickness in 2 key measurements: Non-keratinized epithelium thickness (mean 131.5, SE = 9.94) and Total epithelium thickness (mean 278.1, SE = 12.37), in contrast to Category I, where these measurements were lower (mean 88.3, SE = 5.48 and mean 237.1, SE = 9.40, respectively). In contrast, no significant disparities were observed in stratum corneum thickness and connective tissue width (Table 4).

**Table 4:** Comparison of histological measurements in the layers of the ruminal epithelium (mean ± SD) among rumen samples from Category I (without SARA) and Category II (with SARA) farms

	Category I (n = 20)	Category II (n = 20)	p value	95% confidence interval of the difference
Stratum corneum (µm)	28.8 (10.1)	30.0 (10.7)	0.72	-5.4 to 7.8
Connective tissue (µm)	120.1 (30.2)	116.7 (32.0)	0.73	-23.3 to 16.5
Non-keratinized epithelium (µm)	88.3 (24.5)	131.5 (44.4)	0.001***	20.2 to 66.2
Total epithelium (µm)	237.1 (42.1)	278.1 (55.3)	0.012*	9.5 to 72.5

*Significant difference at p ≤ 0.05

***Significant difference at p ≤ 0.001

### Linear regression analysis

Linear regression analysis between pH_farm_ and values of each color component using CIELab system showed that L had the highest correlation coefficient [r = 0.862 (p < 0.001)], while b had a smaller degree of correlation [r = 0.456 (p < 0.001)]. However, a showed a lower and non-significant degree of correlation [r=-0.160 (p = 0.170)] (Table 5).

**Table 5:** Linear regression analysis of color components (x) and pH_farm_ (y)

Analysis System	Color component	pH_farm_		
		Pearson test [r (p value)]	R^2^	Equation
CIELab	L	0.862 (< 0.001)***	0.743	y=-3.7e^2^ + 70.19x
	a	-0.160 (0.170)		
	b	0.456 (< 0.001)***	0.208	y=-1.67e^2^ + 29.95x
RGB	Red	0.853 (< 0.001)***	0.727	y=-9.08e^2^ + 1.72e^2^x
	Green	0.844 (< 0.001)***	0.713	y=-8.76e^2^ + 1.66e^2^x
	Blue	0.650 (< 0.001)***	0.422	y=-5.69e^2^ + 1.12e^2^x
	RGB-SUM	0.835 (< 0.001)***	0.697	y=-2.35e^3^ + 4.5e^2^x
	HSV	0.402 (< 0.001)***	0.161	y=-2.61e^2^ + 48.46x
	HSL	0.753 (< 0.001)***	0.568	y=-3.27e^2^ + 61.71x

***Significant difference at p ≤ 0.001

L: color lightness

a: redness-greenness of the color

b: yellow-blueness of the color

HSV: Hue, Saturation and Value

HSL: Hue, Saturation, and Lightness

In linear regression analysis between pH_farm_ and each component from RGB color analysis, significant correlations were found between the RGB, RGB-SUM, HSV, and HSL components. Red had the highest degree of correlation [r = 0.853 (p < 0.001)], while Green and RGB-SUM had smaller degrees of correlation [r = 0.844 (p < 0.001) and r = 0.835 (p < 0.001), respectively]. However, HSL, Blue, and HSV had lower degrees of correlation [r = 0.753 (p < 0.001), r = 0.650 (p < 0.001), and r = 0.402 (p < 0.001), respectively] (Table 5).

Non-keratinized epithelium thickness had the highest correlation coefficient [r=-0.778 (p < 0.001)] among the layers of ruminal epithelium, while Total epithelium thickness showed a smaller but significant degree of correlation [r=-0.578 (p < 0.001)]. However, there was no significant correlation coefficient for stratum corneum thickness and connective tissue width (Table 6).

**Table 6:** Linear regression analysis of histological measurements in the layers of the ruminal epithelium (x) and pH_farm_ (y)

	pH_farm_		
	Pearson test [r (p value)]	R^2^	Equation
Stratum corneum (µm)	0.085 (0.604)		
Connective tissue (µm)	0.034 (0.835)		
Non-keratinized epithelium (µm)	-0.778 (< 0.001)***	0.606	y = 1.03e^3^-1.6e^2^x
Total epithelium (µm)	-0.578 (< 0.001)***	0.334	y = 1.12e^3^-1.51e^2^x

***Significant difference at p ≤ 0.001

Among CIELab system components, L showed a high degree of correlation [r=-0.637 (p < 0.001)], while b showed a smaller but significant correlation with non-keratinized epithelium thickness. However, a showed no significant correlation coefficient (Table 7).

**Table 7:** Linear regression analysis of color components (x) and non-keratinized epithelium thickness (y)

Analysis System	Color component	Non-keratinized epithelium (µm)		
		Pearson test [r, (p value)]	R^2^	Equation
CIELab system	L	-0.637 (0.001)***	0.406	y = 1.65e^2^-1.61x
	a	0.248 (0.123)		
	b	-0.464 (0.003)^b^	0.215	y = 1.2e^2^-1.66x
RGB System	Red	-0.634 (< 0.001)***	0.402	y = 1.64e^2^-0.65x
	Green	-0.629 (< 0.001)***	0.395	y = 1.66e^2^-0.68x
	Blue	-0.461 (0.003)*	0.212	y = 1.51e^2^-0.57x
	RGB-SUM	-0.605 (< 0.001)***	0.366	y = 1.65e^2^-0.23x
	HSV	0.086 (0.597)		
	HSL	-0.565 (< 0.001)***	0.319	y = 1.48e^2^-1.4x

*Significant difference at p ≤ 0.05

***Significant difference at p ≤ 0.001

L: color lightness

a: redness-greenness of the color

b: yellow-blueness of the color

HSV: Hue, Saturation and Value

HSL: Hue, Saturation, and Lightness

Similarly, correlation analysis between non-keratinized epithelium thickness and RGB color components revealed that Red had the highest correlation coefficient [r=-0.634 (p < 0.001)], while Green, RGB-SUM, HSL, and Blue had smaller correlation coefficients [r=-0.629 (p < 0.001), r=-0.605 (p < 0.001), r=-0.605 (p < 0.001), and r=-0.565 (p < 0.001), respectively]. However, HSV showed no significant correlation coefficient [r = 0.086 (p = 0.597)] (Table 7).

The correlation coefficient for total epithelium thickness and values of each color component using the CIELab system revealed that L had the greatest correlation coefficient [r=-0.474 (p = 0.002)], while a and b showed no significant correlation (Table 8).

**Table 8:** Linear regression analysis of color components (x) and total epithelium thickness (y)

Analysis System	Color component	Total epithelium (µm)		
		Pearson test [r, (p value)]	R^2^	Equation
CIELab system	L	-0.474 (0.002)*	0.224	y = 3.1e^2^-1.51x
	a	0.137 (0.399)		
	b	-0.255 (0.112)		
RGB System	Red	-0.476 (0.002)*	0.226	y = 3.11e^2^-0.65x
	Green	-0.466 (0.002)*	0.217	y = 3.08e^2^-0.61x
	Blue	-0.392 (0.012)*	0.153	y = 3.02e^2^-0.62x
	RGB-SUM	-0.467 (0.002)*	0.218	y = 3.12e^2^-0.23x
	HSV	0.055 (0.738)		
	HSL	-0.428 (0.006)*	0.184	y = 2.95e^2^-1.35x

*Significant difference at p ≤ 0.05

L: color lightness

a: redness-greenness of the color

b: yellow-blueness of the color

HSV: Hue, Saturation and Value

HSL: Hue, Saturation, and Lightness

Similarly, correlation analysis between total epithelium thickness and RGB color components showed that Red had a high degree of correlation [r=-0.476 (p = 0.002)], while Green, RGB-SUM, HSL, and Blue had smaller correlation coefficients [r=-0.466 (p = 0.002), r=-467 (p = 0.002), r=-0.428 (p = 0.006), and r=-0.392 (p = 0.012), respectively]. However, HSV showed no significant correlation coefficient [r = 0.55 (p = 0.738)] (Table 8).

## Discussion

SARA is defined as increased periods of rumen pH under 5.6–5.8 (Kleen et al. 2003; Krause and Oetzel 2006). The animals in Category II showed a mean ruminal pH value lower than 5.8, indicating the presence of subacute ruminal acidosis (SARA). In contrast, animals in Category I had a mean ruminal pH value above 5.8, indicating the absence of SARA.

Sampling for pH measurements using the Rumen Scoop has generated concerns about its accuracy among veterinary clinicians. Nevertheless, this device has proven its precision in specific research studies, as exemplified by Geishauser et al. (2012), and has consistently delivered reliable results in our clinical practice. Additionally, taking into account animal welfare concerns, it’s crucial to highlight that esophageal tubing stands as the sole non-invasive method for collecting rumen fluid samples. Therefore, for our current research, we have chosen this method, giving precedence to animal welfare considerations.

The mean values of color components, regardless of the evaluation method (RGB or CIELab), were significantly lower in Category II samples, indicating the presence of dark coloration in the ruminal epithelium. This finding was consistent with the results of Alhidary et al. (2016), where a correction of the ratio in lambs resulted in more light-colored epithelium. Other researchers have observed grey to dark ruminal epithelium due to parakeratosis resulting from lowered pH and increased accumulation of volatile fatty acids (VFAs) in rumen fluid (Enemark 2008; Steele et al. 2009, 2011). However, in our study, we observed an increase in non-keratinized rumen epithelium in Category II animals without parakeratosis. The darkening of the rumen epithelium’s color should be attributed to the effect of rumen pH on the keratinized layer of the epithelium.

The L component in CIELab analysis showed a strong correlation with the average pH_farm_ and could potentially be used as an indicator of improper pH range in the rumen. Similarly, Chmiel et al. (2012) reported that the L component had the greatest correlation coefficient for the color and pH of meat, while the b component was determined to have the greatest correlation coefficient by Wulf et al. (1997). In our study, the b component followed with the next highest correlation coefficient (r = 0.456) (Table 5).

Additionally, using the RGB Analysis system, Red presented the highest degree of correlation with the average pH_farm_ and could be used as an indicator for critical pH values in the rumen (Table 5). Similarly, Chmiel et al. (2012) reported that Red presented with the highest correlation coefficient for the color and pH of meat, with V and L color components to follow. However, in our survey, the Green and RGB-SUM components presented with the next highest correlation coefficient (r = 0.844 and r = 0.835, respectively) (Table 5). Therefore, the Green and RGB-SUM components could be used as alternative parameters for the prediction of abnormal deviations in rumen pH.

The animals in Category II had increased non-keratinized epithelium thickness and total epithelium thickness compared to Category I (Table 4). The thickness of the non-keratinized epithelium showed the strongest correlation with the average pH_farm_ (Table 6). It is possible that the lower rumen pH may increase the rate of replacement of the keratinized epithelium, leading to an increase in the non-keratinized epithelium to correspond to the need of producing keratinized layers.

A correlation between the layers of the ruminal epithelium and color components has remained unexplored in existing literature. In the course of our investigation, a significant statistical correlation was established between the L component and both the non-keratinized epithelium and the total epithelium (r= -0.637 and r= -0.474, respectively). Similarly, within the RGB Analysis system, the Red component exhibited the highest degree of correlation with both the non-keratinized epithelium and the total epithelium (r= -0.634 and r= -0.476, respectively). Following closely in correlation coefficient were the Green, RGB-SUM, and L indexes (as shown in Tables 7 and 8). This noteworthy correlation can be attributed to the prolonged impact of rumen pH on both the color of the epithelium and the thickness of the non-keratinized epithelium, as the animals’ diet remained constant throughout the year-long monitoring period. It is anticipated that conducting further research with a larger sample size will serve to reinforce and enhance these observed correlations.

## Conclusions

This study affirms the potential utility of computerized rumen colorimetry in investigating subacute ruminal acidosis (SARA) in beef cattle. The availability of portable computing devices equipped with color analysis software offers a convenient, swift, and dependable means of assessing the chromatic variation in rumen epithelium. This assessment can be conducted during exploratory necropsy or at the abattoir, rendering it a valuable resource for monitoring the effectiveness of management, nutritional, and preventive interventions within a beef farm setting. To fully explore the prospective application of computerized rumen colorimetry as a diagnostic instrument for live animals, further research is warranted, involving the utilization of a gastroscope.
